# System-Level Factors Contributing to Burnout and Professional Well-Being Among Transgender and Gender-Diverse Nurses

**DOI:** 10.1089/heq.2024.0196

**Published:** 2025-05-09

**Authors:** Hyunmin Yu, Celsea Tibbitt, J. Margo Brooks Carthon, Karen B. Lasater, José A. Bauermeister, Matthew D. McHugh

**Affiliations:** ^1^ Center for Health Outcomes and Policy Research, School of Nursing, University of Pennsylvania, Philadelphia, Pennsylvania, USA.; ^2^ Leonard Davis Institute of Health Economics, University of Pennsylvania, Philadelphia, Pennsylvania, USA.; ^3^ Eidos LGBTQ+ Health Initiative, School of Nursing, University of Pennsylvania, Philadelphia, Pennsylvania, USA.

**Keywords:** transgender and gender-diverse, nurses, gender identity, burnout, professional well-being, workplace discrimination

## Abstract

**Introduction:**

Transgender and gender-diverse (TGD) health care professionals face significant burnout, yet evidence on system-level factors such as workplace discrimination that contribute to this issue among TGD nurses is limited. Responding to the U.S. Department of Health and Human Services’ call for research on burnout among health care staff from diverse demographic backgrounds, this study aimed to examine the system-level factors affecting burnout and professional well-being among TGD nurses and identify strategies to enhance their well-being.

**Methods:**

This qualitative descriptive study employed directed content analysis of responses of 6 different open-text survey questions from 66 TGD nurses, utilizing 2 nurse survey datasets collected pre-pandemic (December 2019–February 2020) and during the pandemic (April 2021–June 2021). To identify the system-level factors influencing burnout and well-being, the themes were developed using both inductive and deductive analytic approaches, guided by the Systems Model of Clinical Burnout and Professional Well-being.

**Results:**

Three themes related to the system-level factors affecting burnout and professional well-being among TGD nurses were: (1) *poor working conditions* characterized by inadequate staffing and insufficient protective measures, (2) *lack of leadership support and a non-inclusive culture* highlighted by unsupportive leadership and workplace discrimination, and (3) *non-patient-centered U.S. health care and LGBTQ+ stigma* that create barriers to providing quality care for marginalized groups and receiving care for themselves. Recommendations from participants to enhance their professional well-being are also presented.

**Conclusion:**

To support the well-being of TGD nurses and improve the quality of care for all patients, health care organizations should implement system-level changes that create a physically and psychologically safe and inclusive work environment.

## Introduction

Nurses comprise the largest segment of the health care workforce^1^ and have long struggled with persistent burnout. Prior to the COVID-19 pandemic, about 30% of nurses reported experiencing burnout.^2^ That figure has risen to nearly half of the 4.7 million nurses in the United States today.^3^ This ongoing challenge poses a serious risk to health care safety^4,5^ and leads to negative effects on nurses, including increased job dissatisfaction,^6,7^ as well as adverse patient outcomes, including higher mortality rates.^8–11^

Health equity emphasizes fair access to resources, treatment, and support systems for all individuals, especially marginalized communities.^12^ However, nurse burnout significantly hinders this equity by undermining health care professionals’ ability to provide optimal care.^11,13^ Social determinants of health, such as race and gender, increase burnout risks among nurses, creating a cycle where those most vulnerable to stress may be the least equipped to manage it. Nurses from minoritized backgrounds, particularly racial and ethnic minority nurses, experience higher burnout rates,^14,15^ compromising the quality of care they provide and negatively impacting patient outcomes, ultimately perpetuating health disparities.^11^

In addition to affecting patient outcomes, burnout has serious consequences for nurses’ own health, including increased risks of depression, anxiety, and cardiovascular disease.^16^ These risks may be even higher for transgender and gender-diverse (TGD) nurses, given evidence of disproportionately high levels of social stress and mental health challenges among TGD individuals.^17^ TGD health care professionals report higher burnout than cisgender peers.^18,19^ Qualitative studies of TGD physicians reveal that emotional distress often results from transphobia, a rigid binary gender system, and institutional factors that jeopardize their safety, leading to feelings of isolation. These professionals frequently alter their speech or mannerisms to conceal their TGD identity and often encounter derogatory remarks or witness discriminatory care toward TGD patients.^20,21^ Despite these findings, no literature specifically examines factors contributing to burnout among TGD nurses. This gap is largely because of noninclusive national nursing surveys, such as the National Sample Survey of Registered Nurses, which include only a binary sex question and do not systematically collect data on sexual orientation or gender identity.

Burnout is exacerbated by systemic inequities and discrimination, including interpersonal racism, racial injustice, homophobia, and transphobia, particularly impacting communities of color and lesbian, gay, bisexual, transgender, queer, or other sexual and gender-diverse (LGBTQ+) individuals.^22^ The U.S. Department of Health and Human Services has emphasized the urgent need for research that examines the varying impacts of burnout among health care staff from diverse demographic backgrounds and the role of system-level factors contributing to workplace stress.^22^

### Conceptual framework

The Systems Model of Clinical Burnout and Professional Well-being^23^ integrates theories from human factors, systems engineering, job design, and occupational health and operates across three levels: (1) frontline care delivery, (2) health care organizations, and (3) the external environment. This framework highlights continuous learning and improvement at each level to reduce burnout and improve clinician well-being.

Frontline care delivery represents the “work system” where care team members—clinicians, patients, and families—interact. Organizational conditions and the physical environment influence these interactions. The health care organization, as the second level, encompasses all care-providing entities and includes elements such as organizational culture, leadership styles, and policies. The third level, the external environment, consists of political, professional, and societal influences that shape actions within health care organizations and at the frontline care level. The interactions between these levels and the work system factors (e.g., job demands and resources) directly influence clinician burnout and well-being. These outcomes, in turn, have significant implications for patients, organizations, and society.

In response to the U.S. Department of Health and Human Services’ call for research exploring burnout across health care staff from various demographic backgrounds and guided by the Systems Model of Clinical Burnout and Professional Well-being, this study examined (1) the system-level factors contributing to burnout and professional well-being among TGD nurses and (2) potential strategies to improve their well-being.

## Methods

### Study design

We employed a qualitative descriptive design, which is well-suited for capturing firsthand accounts with minimal interpretation. This approach enables a rich, straightforward description of participants’ experiences while identifying patterns in the data.^24^ Given the limited research on TGD nurses’ workplace experiences, this design provided a structured yet flexible framework to analyze their perspectives. Through this approach, we aimed to gain a detailed understanding of system-level factors affecting their professional well-being and their recommendations for improvement while remaining close to the participants’ own words.

### Data sources

This study is a directed content analysis of open-text responses from registered nurses (RN) who identified as TGD in New York and Illinois. These responses were drawn from two survey datasets collected pre-pandemic (December 2019–February 2020) and during the pandemic (April 2021–June 2021).^25^ The surveys, conducted via email, were large-scale efforts to evaluate nurse demographics, job outcomes, and the quality of nurses’ work environments in both states.^25^ Survey questions included close-ended questions and open-text responses. We explored the open-text responses from both the 2019 and 2021 surveys. To comprehensively examine the system-level factors contributing to burnout and well-being among TGD nurses, as well as the recommended strategies for improvement, we analyzed responses to six different questions that addressed the system-level factors:

- Is there anything else you would like to share about your experiences with stress in the workplace (e.g., burnout, racism, violence)?
- What would you like to see your hospital do to improve your mental health and well-being?
- What would most improve quality of care in your setting?
- What helps (or hinders) your ability to provide quality care to vulnerable populations?
- Is there anything else you would like to share about your career as a nurse?
- For those who indicated that they would not recommend their hospital as a good place to work, please tell us why not.

### Study sample

To describe factors contributing to burnout and well-being among TGD nurses, we included participants identifying as TGD from the 2019 and 2021 surveys. The gender question in both surveys, “What is your gender?” provided three options: “Man,” “Woman,” and “Other (please specify).” Participants who selected “Other” could specify their gender. After reviewing all written responses, we identified 22 TGD nurses in the 2019 survey and 44 in the 2021 survey, with specified identities including “Transgender,” “Non-binary,” “Genderqueer,” “Agender,” “Gender nonconforming,” and “Pangender.” While there was no identifier to confirm whether any of these participants were the same across both surveys, we confirmed 66 distinct TGD nurses based on demographic characteristics (e.g., age, race, ethnicity, entry-level nursing education). The study protocols were approved by the Institutional Review Board, and all participants provided informed consent, which included permission for their de-identified responses to be used in future research. Therefore, re-contacting or re-consenting participants was not required for this secondary qualitative data analysis.

### Data analysis

Open-ended responses from 66 TGD nurses were analyzed using directed content analysis, which is particularly suited for theory-guided studies and allows researchers to focus on specific aspects of the content.^26–28^ This method enables the identification and categorization of key themes by concentrating on the characteristics of language as a form of communication while also uncovering the contextual meaning behind the text. It emphasizes how language conveys concepts relevant to the study’s objectives, ensuring that the interpretation aligns with the theoretical framework.^26–28^

All responses were uploaded to NVivo version 14 for systematic data encoding. Initially, the first coder (H.Y.) identified 96 content-driven codes through a comprehensive data review and developed a preliminary codebook focused on (1) system-level factors contributing to burnout and professional well-being and (2) recommended strategies to enhance well-being. The codebook included detailed codes, definitions, and examples to guide data allocation. The codebook was then refined in collaboration with a second coder (C.T.) to ensure accuracy, resulting in a final set of 30 codes. Both coders jointly coded 25% of all responses to establish alignment. They then independently applied the codebook to the entire dataset. Intercoder agreement was achieved through consensus, with any coding discrepancies resolved via discussion during weekly meetings.^29,30^ After coding, both coders independently reviewed the coded excerpts and then convened to discuss and refine the emerging themes, selecting key themes and exemplary quotes collaboratively.

### Rigor and trustworthiness

Rigor and trustworthiness were maintained through credibility, dependability, and transferability.^31^ Credibility was enhanced by analyzing responses from TGD nurses across various roles, settings, and regions, capturing diverse perspectives, supported by rich participant quotes. Dependability was ensured through a systematic coding process, including a detailed codebook and collaborative coding to maintain consistency with the study’s aims. Transferability was strengthened by providing comprehensive descriptions of the study context, methods, and participant characteristics, enabling readers to evaluate the findings’ applicability to similar contexts.

### Reflexivity and positionality

Two coauthors, H.Y. and C.T., led the data analysis. H.Y., a male nurse practitioner and postdoctoral research fellow focusing on LGBTQ+ health equity research, brought both professional and personal insights into TGD health care challenges. C.T., a female nurse and postdoctoral research fellow focused on maternal health equity and Black birthing people, contributed a perspective on systemic inequities and intersectionality. Both engaged in reflexivity throughout the process, holding regular debriefing sessions to reflect on potential biases, ensuring the voices of TGD nurses were authentically represented and minimizing the influence of preconceived notions.

## Results

Participant characteristics are detailed in Table 1. Among the 66 TGD nurses, most identified as non-binary (47%), followed by transgender (29%). The majority were White (70%) and non-Hispanic (94%). The average age was 37 years, with 8 years of nursing experience. Most participants held a baccalaureate degree (52%). The majority worked in direct patient care (77%), and nearly half (47%) were employed in a hospital setting.

**Table 1.** Characteristics of Transgender and Gender-Diverse Nurse Participants

Characteristics	Respondents (*n* = 66)
Gender identity (*n* [%])	
Transgender	19 (28.8)
Non-binary identities	31 (47.0)
Gender-diverse identities^a^	16 (24.2)
Age (mean [SD])	37.0 (10.5)
Race (*n* [%])	
White	46 (69.7)
Asian	10 (15.2)
Black	1 (1.5)
Multiracial/Other	9 (13.6)
Ethnicity (*n* [%])	
Hispanic	4 (6.1)
Non-Hispanic	62 (93.9)
State of employment (*n* [%])	
Illinois	24 (36.4)
New York	42 (63.6)
Years of experience (mean [SD])	8.1 (8.0)
Highest nursing degree (*n* [%])	
Associate degree	12 (18.2)
Baccalaureate degree	34 (51.5)
Graduate degree	20 (30.3)
Role (*n* [%])	
Direct patient care	51 (77.3)
Non-patient care	15 (22.7)
Work setting (*n* [%])	
Hospital setting	31 (47.0)
Non-hospital health care setting	24 (36.4)
Non-health care setting	11 (16.6)

^a^ Due to privacy concerns, categories with an extremely small number of participants were merged. This merged category includes genderqueer, gender nonconforming, agender, and pangender identities.

SD, standard deviation.

### System-level burnout drivers among TGD nurses

Table 2 outlines three main themes at each level within the Systems Model of Clinical Burnout and Professional Well-being: (1) *poor working conditions* at the frontline care delivery level, (2) * lack of leadership support and a non-inclusive culture* at the health care organization level, and (3) *non-patient-centered U.S. health care and LGBTQ+ stigma* at the external environment level. In addition, Table 2 includes subthemes (system-level factors contributing to burnout among TGD nurses) and their definitions under each main theme, along with key quotes from participants.

**Table 2.** Summary of Major Qualitative Themes

Theme	Subtheme	Subtheme definition	Key quotation
I. Frontline care delivery (e.g., local organizational conditions, technologies, physical environment, and activities)			
Poor working conditions	Inadequate staffing levels	Inadequate staffing levels lead to increased workload for existing staff, compromised patient care quality, and heightened stress and burnout among TGD nurses.	“Staffing shortages with PCTs [patient care technicians] contribute to significant stress and burnout.” [RN 57] “Our unit is not hiring enough auxiliary staff, and those who remain are burnt out. Caring for 7–8 high-acuity telemetry patients is irresponsible and dangerous. We have a high fall rate because there aren’t enough auxiliary staff. We are constantly asked to do overtime, and we are run ragged.” [RN 33] “In my ICU [intensive care unit], we don’t have CNAs [certified nursing assistants], so it is very hard to provide direct care when everyone is busy.” [RN 50]
	Insufficient protective measures	Insufficient protective measures include limited access to protective equipment and inadequate training or support systems for managing aggressive patients, contributing to trauma and burnout among TGD nurses.	“When confused patients become aggressive, we, as nurses, often find ourselves having to manage the situation alone.” [RN 58] “I continue to grieve the loss of my role as an advanced practice nurse. I am still very angry that I was not protected from COVID; my workplace provided no PPE [personal protective equipment] or patient restrictions, and then laid me off after I became ill.” [RN 55]
II. Health care organization (e.g., leadership and management, governance, organizational rewards and benefits)			
Lack of leadership support and a non-inclusive culture	Unsupportive leadership	Unsupportive leadership and management refer to a lack of support, understanding, and action from those in positions of authority within a health care organization, including dismissive attitudes toward concerns raised by staff, failure to address workplace issues, and a lack of support for employees experiencing burnout or trauma.	“Burnout is linked to my lack of control and power over the policies that affect my job and health, as well as the staffing and policies that contribute to poor patient outcomes.” [RN 44] “Raising a concern with leadership often leads to recrimination and retribution.” [RN 6]
	Unfair treatment and workplace discrimination	Unfair treatment and workplace discrimination refer to experiences of bias, prejudice, and unequal treatment based on a person’s gender identity, sexual orientation, mental health status, or other marginalized characteristics. This can include denial of accommodations, discrimination in promotion processes, and a hostile work environment.	“My burnout stems from the bias and unfair treatment in my work area. I feel that I am being discriminated against.” [RN 62] “I had to leave a previous job due to mental illness. When I requested workplace accommodations, they were denied. After disclosing my mental illness, I was met with disrespect, suspicion, and stigma.” [RN 64] “If you belong to a marginalized group, the promotion process is different. Discrimination continues to persist while it is not as overt as in the 1990s when individuals could be jailed for being transgender.” [RN 51] “I recently discovered that I am part of the LGBTQ+ community. One of the factors contributing to my stress is the inability to be my authentic self at work.” [RN 57] “What stresses me is that our employee pool does not reflect the patient populations we take care of; we have almost no nurses of color.” [RN 30]
III. External environment (e.g., health care industry, laws, regulations, standards, and societal values)			
Non-patient-centered U.S. health care and LGBTQ+ stigma	Barriers to quality care for marginalized groups	Barriers to accessing high-quality care for marginalized groups include language obstacles, limited access to specialized services, discrimination from health care providers, and systemic issues like poverty and lack of insurance. Caring for this population without support contributes to burnout and stress.	“Burnout is exacerbated by seeing the ways in which capitalism makes my already vulnerable patients sicker, with less access to preventative care. I can’t do anything about it. It is disheartening to witness a healthcare system that throws away poor people and sees their chronic diseases as inevitable.” [RN 44] “I work in a predominantly Latinx area where Spanish is a major language. While I am still in the process of learning the language myself, the hospital needs to implement a better system to serve this population.” [RN 50] “Unfortunately, within our current capitalistic and patriarchal healthcare system, many nurses have become hardened, burned out, morally injured, and addicted. This is truly a tragedy.” [RN 29]
	Social exclusion of LGBTQ+ individuals	Social exclusion of LGBTQ+ individuals refers to the isolation, discrimination, and marginalization they experience within health care settings and society, including the nursing profession.	“I feel saddened by my inability to provide gender-affirming care due to my work in the Catholic hospital system, where reproductive rights are often ignored.” [RN 51] “There are insufficient educational opportunities focused on LGBTQ+ and transgender health.” [RN 57]

TGD, transgender and gender-diverse.

### Theme 1 (frontline care delivery): Poor working conditions

This overarching theme emphasizes how poor working conditions within health care contribute to burnout among TGD nurses.

#### Inadequate staffing levels

Participants reported that inadequate staffing levels heightened workloads, compromising the quality of patient care and leading to increased stress and burnout. With fewer nurses and ancillary staff (e.g., nursing assistants) available, the demands of patient care became increasingly challenging to meet.

Due to inadequate staffing, my typical patient load as an RN was between 7 to 9, often exceeding that number. This wasn’t just an occasional occurrence; it happened every shift. Many of our nurses appear visibly shaken and overwhelmed during their shifts, sometimes even in tears from the mountain of critical work required in the ER [emergency room]. My health has been significantly affected by missed breaks and exhausting patient caseloads. [RN 39]

#### Insufficient protective measures

Participants reported frequently facing a lack of access to protective measures and support systems for managing violent patients and their families. This deficiency created an environment that was both physically and emotionally unsafe. The absence of support mechanisms heightened the risk of traumatic encounters, resulting in feelings of helplessness and contributing to burnout.

Being assaulted by patients is becoming too common. Administration does not care and lacks effective solutions. [RN 43]

### Theme 2 (health care organization): Lack of leadership support and a non-inclusive culture

This overarching theme underscores the crucial impact of leadership dynamics and organizational culture on the well-being of TGD nurses.

#### Unsupportive leadership

Participants identified unsupportive leadership as a system-level factor contributing to their burnout. They reported a lack of support, understanding, and action from health care authorities, often experiencing dismissive attitudes toward their concerns, inadequate responses to workplace issues, and insufficient support for employees facing burnout or trauma.

I am an abortion provider, and our clinic was targeted in a shooting this past weekend. I was already experiencing burnout before the pandemic, and it has only worsened since then. As outpatient care slowly returns to “normal,” despite the ongoing pandemic, my burnout continues to escalate and is negatively affecting my health and my family’s well-being. This week, following the violence at our clinic, I have felt especially drained, stressed, and overwhelmed. Upper management has been dismissive and unsupportive during this period of increased violence and trauma. [RN 24]

#### Unfair treatment and workplace discrimination

Participants highlighted that unfair treatment and workplace discrimination significantly impacted their well-being. They reported experiencing bias and unfair treatment based on gender identity, sexual orientation, and mental health status, leading to feelings of unrecognized and disrespectful treatment in their work environments. These experiences, along with feeling unsafe to speak up or report them, contributed to burnout.

There has been very little consideration for me as a queer and trans person. This causes stress in everyday interactions and has increased burnout for me. [RN 66]

### Theme 3 (external environment): Non-patient-centered U.S. health care and LGBTQ+ stigma

This overarching theme highlights the significant impact of societal influences and systemic barriers on the experiences of TGD nurses.

#### Barriers to quality care for marginalized groups

Participants emphasized barriers that hindered access to high-quality care for marginalized populations, negatively affecting patient outcomes and their well-being as health care providers. These barriers included insufficient support for patients with limited English proficiency or psychiatric disorders, restricted access to specialized services (e.g., abortion clinics), experiences of systemic racism and transphobia, and broader issues, including poverty and lack of insurance. Navigating these challenges without adequate support contributed to burnout.

Caring for psychiatric patients under the current healthcare system is nearly impossible. The system is a revolving door of medications, drugs, homelessness, and poverty, offering little hope. There is a lack of long-term psychiatric care, and the work can be dangerous, with inexperienced staff often being the first point of contact for complex behavioral health patients. While I am passionate about caring for these individuals, I was simply providing “treat and street.” This is stressful. [RN 48]

#### Social exclusion of LGBTQ+ individuals

Participants emphasized that the social exclusion of LGBTQ+ individuals, particularly in nursing, profoundly impacted their ability to deliver individualized care to their patients and to receive appropriate care for themselves. Many expressed frustration and helplessness due to institutional barriers that hindered their understanding of transgender health and rights and their capacity to provide inclusive care, such as gender-affirming services. This exclusion contributed to a lack of awareness regarding the unique health care needs of TGD patients and staff.

Nursing is exceptionally exclusionary, exhibiting racism, homophobia, transphobia, transmisogyny, and anti-queer sentiments. [RN 27]

### TGD nurses’ recommended solutions for their burnout

Table 3 presents TGD participants’ recommendations for enhancing the professional well-being of their peers. TGD nurses emphasized the need for improved nurse-to-patient ratios, adequate ancillary staff, and a safe work environment within the frontline care delivery domain. Within the health care organization domain, they advocated for transparent communication between administration and staff, shared decision-making with frontline nurses, managers who listen and act on concerns, and an inclusive organizational culture that values workforce diversity. In the external environment domain, they stressed the importance of system support for marginalized patients, including effective language services and community resources, as well as open discourse on TGD health and rights in nursing practice.

**Table 3.** Recommendations from Transgender and Gender-Diverse Nurses to Improve the Professional Well-Being of Their Peers

Recommendation	Key quotation
I. Frontline care delivery (e.g., local organizational conditions, technologies, physical environment, activities)	
Improved nurse-to-patient ratio and sufficient ancillary staff	“Improving the nurse-to-patient ratio is essential to reducing burnout and stress levels at work.” [RN 36] “Enough staffing, thorough training of staff with efficient nursing educators, and enough ancillary like CNAs [certified nursing assistants], are essential for us to perform our jobs effectively.” [RN 1]
Safe work environment	“A physical environment that I feel I can depend on.” [RN 34] “Focus on staff safety.” [RN 55]
II. Health care organization (e.g., leadership and management, governance, organizational rewards and benefits)	
Transparent communication	“I recommend more transparency between management and staff.” [RN 12] “Improve communication between teams in the hospital and administrators.” [RN 58]
Shared decision-making with frontline nurses	“Take our feedback and consider us when making business and policy decisions.” [RN 66] “More frontline staff representation in leadership spaces.” [RN 24] “Nurses providing care need to be involved in decision-making about the unit.” [RN 41]
Listening and taking action to concerns	“We need an administrative team that listens to concerns.” [RN 52] “I want managers who listen to rather than stifle our cries for help.” [RN 6]
Inclusive culture and workforce diversity	“Take action against workplace discrimination.” [RN 50] “We need more staff of color and more transgender and non-binary staff.” [RN 24]
III. External environment (e.g., health care industry, laws, regulations, and standards, societal values)	
System support for marginalized patients	“The effective language line will be helpful for providing care to non-English-speaking patients.” [RN 41] “We need effective means in providing community resources for people with economic instability.” [RN 62] “Hospitals need to create a patient-centered environment that focuses more on improving long-term patient outcomes and less on volume of patients served.” [RN 58]
Discourse around transgender health and rights in nursing	“More active and effective nursing discussion focused on diversity among both coworkers and patients.” [RN 57] “Talk about abortion rights and transgender rights.” [RN 24]

## Discussion

Our research addresses a critical gap in contextualizing how systemic structures and dynamics influence burnout among TGD nurses. Guided by the Systems Model of Clinical Burnout and Professional Well-being,^23^ we identified system-level factors known to contribute to nurse burnout, as well as those that may have a differential impact on this specific population.

Our findings show that TGD nurses face work-related challenges similar to those reported among nurses more broadly, including poor working conditions and unsupportive leadership. Consistent with prior studies,^32,33^ inadequate staffing levels emerged as a prominent concern, with participants reporting overwhelming patient loads that hindered their ability to provide quality care. The stress of juggling multiple responsibilities in an under-resourced environment not only impacts nurses’ burnout but also compromises the quality of patient care, creating a vicious cycle of stress and burnout.^8,32^ The emotional toll of these conditions, including rising workplace violence against nurses,^34^ is exacerbated by insufficient protective measures, leaving TGD nurses exposed to both physical and emotional risks. Systemic changes, including improving staffing ratios and ensuring access to protective resources, are essential for creating a safer, more supportive environment that enhances the well-being of both nurses and patients. Similarly, unsupportive leadership emerged as a key contributor to burnout among TGD nurses, aligning with previous research.^35,36^ Many participants felt neglected, reporting that their concerns were often disregarded and that they lacked involvement in key policy decisions. Leadership’s failure to communicate transparently exacerbated frustration, fostering an organizational culture that perpetuates burnout.

Our findings also highlight unique challenges faced by TGD nurses. Participants shared instances of unfair treatment and discrimination based on gender identity and sexual orientation, as well as a lack of psychological safety when reporting or discussing such experiences. Although a direct comparison of our findings to other literature on TGD nurses’ experiences is not possible due to the lack of existing research, similar discriminatory experiences, such as being misgendered, excluded from professional networks, and facing hostility from colleagues and patients, have been documented among other health care professionals, including physicians.^18,19^ These findings underscore a broader organizational climate that often neglects diversity and inclusivity, further marginalizing TGD nurses in the workplace. This is particularly concerning in the context of current federal attacks on diversity, equity, and inclusion initiatives, as well as increasing threats to TGD health, rights, and research.^37^ The lack of institutional commitment to fostering an inclusive and supportive work environment not only exacerbates the challenges faced by TGD nurses but may also have downstream effects on workforce retention and the quality of care provided to diverse patient populations.

To improve the well-being of TGD nurses, health care organizations should prioritize supportive and inclusive leadership. Nurse leaders in a recent study suggested various strategies to create a more LGBTQ+ inclusive environment.^38^ Some of these recommendations aligned with strategies suggested by TGD nurses in this study. First, fostering a flexible organizational culture through transparent communication is essential. Second, promoting workforce diversity is beneficial, as it brings varied perspectives that enhance decision-making. Most participants emphasized the importance of having staff that reflects the broader community, including LGBTQ+ individuals, to better understand and address their needs. In addition, embedding inclusivity into institutional policies, including implementing comprehensive LGBTQ+ training programs for both leadership and staff,^39^ is crucial.

Our findings emphasize the impact of societal influences and systemic barriers on TGD nurses’ experiences. Many participants reported working in health care systems that lack a patient-centered approach, limiting their ability to provide effective care for marginalized populations. Consistent with previous research on Black nurses’ experiences,^14^ systemic barriers, including inadequate resources for marginalized groups, not only harm patient outcomes but also exacerbate the emotional burden on minoritized nurses. This underscores the need for reforms prioritizing equity and access in health care. Policymakers must advocate for more inclusive practices and allocate resources to better support marginalized communities, while participants also called for ongoing advocacy efforts within the nursing profession regarding TGD rights and health.

Burnout among TGD nurses affects both their well-being and patient outcomes.^11,13^ The Systems Model of Clinical Burnout and Professional Well-being underscores the need for system-level interventions beyond individual-focused strategies.^23^ Continuous improvement across practice, education, and research is essential for fostering a supportive and inclusive work environment. Health care organizations should implement TGD-inclusive employee assessments and provide safe spaces for sharing experiences to enhance organizational learning and well-being. LGBTQ+ health content and inclusive care strategies should both be integrated into curricula and continuing education. Researchers should prioritize inclusive data collection, including comprehensive sex and gender measures,^40^ to better examine the intersection of gender identity, workplace dynamics, and burnout. A multisectoral effort is crucial to ensuring that TGD nurses feel valued, supported, and empowered to provide high-quality care.

The strengths of this study lie in its theory-informed qualitative analysis of burnout factors affecting an underrepresented population. However, several limitations exist. First, although our survey represents a critical advancement beyond the binary sex categories commonly used in national nursing surveys, its gender question was not fully comprehensive. For example, transgender women may have selected “Woman” instead of specifying “Transgender,” potentially underrepresenting TGD identities in the dataset. Future research should assess sex and gender separately to ensure inclusivity.^40^ Second, our sample from two states with relatively progressive LGBTQ+ stances may not accurately reflect the broader TGD nursing population. Future studies should recruit TGD nurses from regions with more entrenched discrimination to effectively address TGD health issues. In addition, the predominance of White participants may limit our findings’ representation of the intersectional dimensions of burnout experiences. Lastly, while we compared participants from the 2019 and 2021 surveys, some nurses may have participated in both. Despite these limitations, this study enhances understanding of burnout among TGD nurses and underscores the need for more inclusive research methodologies in nursing.

## Conclusions

To support the well-being of TGD nurses and improve the quality of care for all patients, health care organizations should implement systemic changes that create a physically and psychologically safe and inclusive work environment.

## Data Availability

The datasets used and/or analyzed during the current study are not available.

## Abbreviations Used

CNA: Certified nursing assistant
COVID-19: Coronavirus disease 2019
ICU: Intensive care unit
LGBTQ+: Lesbian, gay, bisexual, transgender, queer, or other sexual and gender-diverse individuals
PCT: Patient care technician
PPE: Personal protective equipment
RN: Registered nurse
SD: Standard deviation
TGD: Transgender and gender-diverse
U.S.: United States
